# Microparticles Engineered to Highly Express Peroxisome Proliferator-Activated Receptor-γ Decreased Inflammatory Mediator Production and Increased Adhesion of Recipient Monocytes

**DOI:** 10.1371/journal.pone.0113189

**Published:** 2014-11-26

**Authors:** Julie Sahler, Collynn F. Woeller, Richard P. Phipps

**Affiliations:** 1 Department of Microbiology and Immunology, School of Medicine and Dentistry, University of Rochester, Rochester, United States of America; 2 Department of Environmental Medicine, School of Medicine and Dentistry, University of Rochester, Rochester, United States of America; IRCCS Istituto Oncologico Giovanni Paolo II, Italy

## Abstract

Circulating blood microparticles are submicron vesicles released primarily by megakaryocytes and platelets that act as transcellular communicators. Inflammatory conditions exhibit elevated blood microparticle numbers compared to healthy conditions. Direct functional consequences of microparticle composition, especially internal composition, on recipient cells are poorly understood. Our objective was to evaluate if microparticle composition could impact the function of recipient cells, particularly during inflammatory provocation. We therefore engineered the composition of megakaryocyte culture-derived microparticles to generate distinct microparticle populations that were given to human monocytes to assay for influences recipient cell function. Herein, we tested the responses of monocytes exposed to either control microparticles or microparticles that contain the anti-inflammatory transcription factor, peroxisome proliferator-activated receptor-γ (PPARγ). In order to normalize relative microparticle abundance from two microparticle populations, we implemented a novel approach that utilizes a Nanodrop Spectrophotometer to assay for microparticle density rather than concentration. We found that when given to peripheral blood mononuclear cells, microparticles were preferentially internalized by CD11b+ cells, and furthermore, microparticle composition had a profound functional impact on recipient monocytes. Specifically, microparticles containing PPARγ reduced activated monocyte production of the proinflammatory cytokines interleukin-8 and monocyte chemotactic protein-1 compared to activated monocytes exposed to control microparticles. Additionally, treatment with PPARγ microparticles greatly increased monocyte cell adherence. This change in morphology occurred simultaneously with increased production of the key extracellular matrix protein, fibronectin and increased expression of the fibronectin-binding integrin, ITGA5. PPARγ microparticles also changed monocyte mRNA levels of several genes including those under PPARγ control. Overall, the delivery of PPARγ from microparticles to human monocytes influenced gene expression, decreased inflammatory mediator production and increased monocyte adherence. These results support the concept that the composition of blood microparticles has a profound impact on the function of cells with which they interact, and likely plays a role in vascular inflammation.

## Introduction

Microparticles are submicron vesicles that are released from the plasma membranes of blood cells and range in density from 5–50 µg per mL of blood plasma [1]. Among the cell types that generate microparticles, platelets and megakaryocytes produce around 80% of blood microparticles [1], [2]. Microparticles are formed during membrane vesiculation induced by activation or apoptosis, and they contain surface receptors, lipids, RNA and proteins derived from their parent cell [3]. Microparticles can signal surface receptors, or even transfer lipids and surface proteins to recipient cells to impact their function. However, little is known about the specific effects of microparticle internal composition on transcellular communication. As a whole, circulating microparticles are now widely recognized to participate in vascular inflammation and thrombosis [1], [3]–[5]. Numerous inflammatory diseases such as arteriosclerosis, diabetes, cancer, sepsis, acute coronary syndromes and others demonstrate elevated microparticle numbers [3]. Additionally, microparticle cell sources are often different in inflammatory conditions [6]. A major knowledge gap remains regarding how the different composition of microparticles would alter the function of recipient cells. The fact that hundreds of mediators can differ between microparticle populations causes a conundrum when trying to compare influences from microparticles with different compositions. We aimed to control variables between microparticle groups by generating two similar microparticle populations that stem from the same cell line. Another important question is to determine which blood cells of a mixed cell population show preference to interact with and internalize microparticles.

The existing literature focuses on how the microparticle source and surface marker composition participate in transcellular communication. However, there is little information regarding the internal composition of microparticles and how this affects recipient cell function. Our lab was the first to discover that the anti-inflammatory transcription factor, peroxisome proliferator-activated receptor-γ (PPARγ) was present in normal megakaryocytes, platelets and platelet microparticles [7], [8]. To determine if the levels of PPARγ in microparticles could impact the function of recipient cells, we first established a platform technology to engineer and isolate microparticles from a human megakaryoblastic cell line (Meg-01) [9]. Meg-01 cells were chosen because they spontaneously and robustly produce microparticles containing undetectable levels of PPARγ. When Meg-01 cells are transduced to highly express PPARγ, their microparticles also contain high levels of PPARγ. This technique served to generate two comparable microparticle groups whose only engineered difference was PPARγ expression levels. We previously established that these microparticles could be internalized by a monocytic cell line, THP-1 and induced the synthesis of fatty acid binding protein-4 (FABP4) [9].

In this work, we wanted to understand whether or not the presence of PPARγ in microparticles influences recipient blood cell function, particularly during an inflammatory provocation. To begin, we first identified mRNA profile differences of the engineered microparticles to determine if PPARγ-containing microparticles had altered mRNA composition. Subsequently, we sought to evaluate if these microparticles could be taken up by primary blood cells, and if so, what subsets preferentially internalized them. Ultimately, we wanted to determine if microparticles could differentially influence recipient cell function via changes in 1) the recipient blood cell transcriptome, 2) cytokine responses to inflammatory stimuli, and 3) other changes indicative of altered function. We hypothesized that treatment with microparticles which contain PPARγ would decrease inflammatory responses of recipient cells. This research will show the first solid evidence that internal microparticle composition can functionally impact recipient cells, and additionally will show the profound impact of PPARγ in transcellular communication.

## Materials and Methods

### Cells and culture conditions

Peripheral human mononuclear cells (PBMCs) were obtained via venipuncture from consenting healthy human donors with written consent in accordance with the Declaration of Helsinki and approved by the University of Rochester Institutional Review Board. Briefly, the buffy coat was separated and diluted in phosphate buffered saline (PBS; Sigma -Aldrich Co., St Louis, MO). PBMCs were isolated using Ficoll-Paque (Amersham Biosciences, Piscataway, NJ) gradient centrifugation. Monocytes were then purified from the leukocyte layer using CD14 Dynabeads (Invitrogen, Carlsbad, CA). CD14 Dynabead-cell rosettes were disrupted using the provided release buffer (Invitrogen). Cells obtained by this method of isolation were >98% CD14^+^.

The cell lines Meg-01 and THP-1 were obtained from American Type Culture Collection (ATCC, Rockville, MD, USA). All cells were cultured in RPMI-1640 (Invitrogen) with 5% Hyclone heat-inactivated fetal bovine serum (Thermo Fisher Scientific, Waltham, MA, USA), 10 mM Hepes (Sigma), 2 mM L-glutamine (Invitrogen), 4.5 g/L glucose (Invitrogen), and 50 µg/mL gentamicin (Invitrogen).

Meg-01 cells or THP-1 cells were transduced with either a control GFP-expressing virus or a GFP and PPARγ-expressing virus [10] at a ratio of 20 MOI (20 transducing units per cell). Cell cultures were expanded and efficiency was verified by flow cytometry. Only Meg-01 cultures that had greater than 90% GFP expression were used to make microparticles. New transductions of naïve Meg-01 cells were performed at least bimonthly to maintain high GFP expression percentages of Meg-01 cultures and subsequent microparticles.

### Microparticle generation and isolation

150 mL of tranduced Meg-01 cells were grown in fresh media for 72 hours before spontaneously generated microparticles were harvested. This was performed via room temperature serial centrifugation as previously described [9]. Briefly, cells were removed via centrifugation at 200×g for 15 minutes. Subsequently, platelet like particles, cell debris, and other large particles were removed by spinning the supernatant at 1000×g for 10 minutes, and then 3000×g for 15 minutes. After each centrifugation, the supernatant was carefully moved to a new centrifuge tube before the next spin. Finally, microparticles were pelleted at 10,000×g for 60 minutes, supernatant was thoroughly aspirated, and the microparticle pellets were resuspended in 400 µL serum-free RPMI1640 if used for treatment of target cells, or with complete lysis buffer (2% SDS, 50 nM Tris-HCL, pH 6.8, Protease Inhibitor (Sigma, P8340) if used for western blots, or with Qiazol (Qiagen) if used for qPCR analysis.

### Microparticle relative concentration determination and treatment of target cells

When culture-derived microparticles were given to cultured cells, microparticles were isolated (see above) and resuspened in 400 µL serum-free RPMI1640. A 5 µL sample was transported for non-sterile quantification in order to leave the remainder of the sample sterile for future culture. To quickly normalize quantities of microparticles from different Meg-01 cultures, a novel approach was developed in where 2 µL of resuspended microparticles were loaded onto a Nanodrop 1000 Spectrophotometer that was previously blanked on RPMI1640 alone, and absorbance was read at 280 nm. The concentration given to microparticles was the same as the calculated protein concentration, but units were denoted as arbitrary (AU) due to the fact that microparticle suspensions are not uniquely protein. Importantly, this value of microparticle concentration is not microparticle numbers/volume, rather microparticle absorbance/volume that represents the relative microparticle concentration for accurate comparison of the two experimental populations of microparticles. The nanodrop spectrophotometer was more sensitive and generated a more accurate linear relationship during measurements of serial diluted microparticles compared to the previously used BCA assay [9] to measure microparticle concentrations (Figure S1). Because it was determined that the AU microparticle concentration could be serially diluted in a linear relationship with absorbance, relative microparticle concentrations could be appropriately calculated and proportionally diluted to achieve equal microparticle concentration between parallel populations. During experiments that compared influences of recipient cells exposed to different microparticle groups, equal levels of Control or PPARγ microparticles were added to recipient cells in a ratio of 20 AU microparticles per 10,000 recipient cells.

Recipient cells were left unactivated or activated with an indicated stimulant using the following concentrations: 50 ng/mL phorbol-12-myristate-13-acetate (PMA; Sigma-Aldrich), 100 ng/mL LPS (Sigma L6529), or 10 ng/mL PAM3CSK4 (IMG-2201, Imgenex, San Diego, CA).

### Microscopy

Live THP-1 cells were examined in culture dishes by a phase-contrast Olympus IX81 inverted microscope. Fixed cytospun cells and fixed cells grown in chamber slides were analyzed with differential interference contrast on an Olympus BX51 light microscope (Olympus, Melville, NY, USA). Images were captured and edited with SPOT RT 5 (Diagnostic Instruments Inc., New Hyde Park, NY, USA).

### Oil-Red-O Staining

THP-1 cells were treated in wells on a 8-well chamber slide (Millipore, Billerica, MA), washed twice with PBS, fixed in 3% paraformaldehyde (Electron Microscopy Sciences, Hatfield, PA), washed again in PBS and then covered with diluted Oil-Red-O [60% Oil Red O in isopropanol diluted in water; 0.2 µm filtered] (Cayman Chemical Company, Ann Arbor, Michigan). The solution was incubated on a rotating rocker for 10 minutes, washed twice with distilled water, mounted with coverslip and then images were taken using differential interference contrast microscopy.

### Dynamic Light Scattering

**Figure 1:**
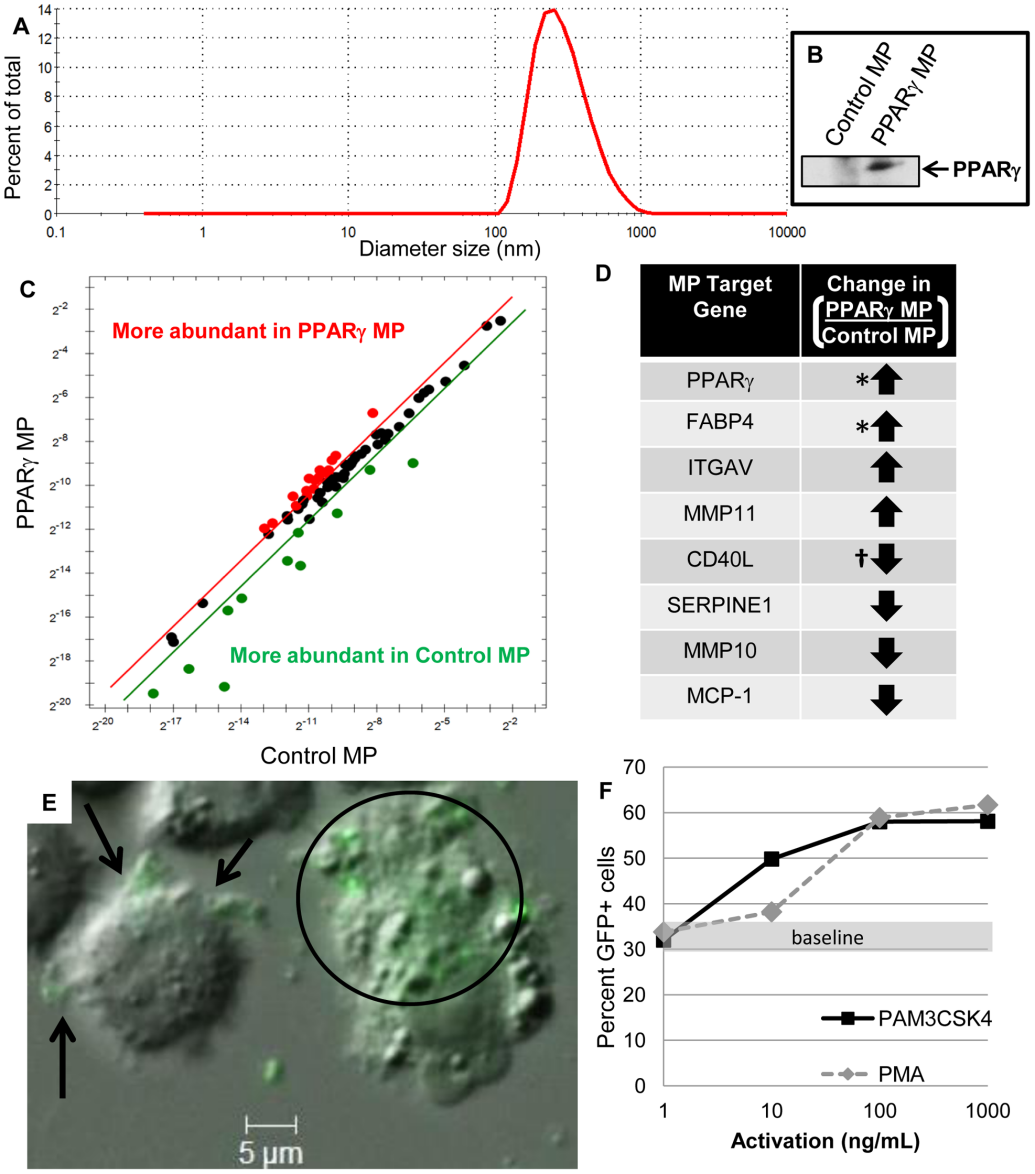
Culture-derived microparticles engineered to contain PPARγ are internalized by THP-1 cells. Microparticles isolated from Meg-01 cultures were characterized for size by dynamic light scattering. **A**, Percent of the total number of analyzed particles was graphed showing the engineered microparticles have diameters ranging from 0.1–1 µm. **B**, Control MP (1) or microparticles that contain PPARγ (PPARγ MP; 2) were lysed and analyzed via western blot for PPARγ protein expression (arrow). **C**, Expression of mRNA transcripts were compared between Control MP or PPARγ MP, where red dots indicate transcripts that are more abundant in PPARγ MP, and green dots indicate transcripts that are more abundant in Control MP. **D**, Select transcript targets with arrows highlight comparative differences between the microparticle groups. * signifies transcripts were undetectable in Control MP, **^†^** signifies transcripts that were undetectable in PPARγ MP. **E**, Green fluorescent protein (GFP)-positive microparticles were added to THP-1 culture for six hours before the cells were collected, fixed, cytospun, and visualized by microscopy. Microparticles (green) are associated with the cell surface (arrows) or within the cytoplasm (circle). **F**, Microparticle internalization by THP-1 cells with sub-saturating numbers of microparticles was measured by flow cytometry and graphed. Basal microparticle internalization rose from approximately 33% of cells (grey horizontal bar) to 60% with increasing doses of PAM3CSK4 (black solid line) or PMA (grey dash line).

Microparticles were isolated from 150 mL culture supernatant, resuspended in 1 mL PBS, and further diluted 1∶10 and mixed thoroughly via pipette immediately before analysis on the Nano ZS Zetasizer (Malvern, Westborough, MA), with an average approximate count rate of 400 kcps. A representative histogram of number-based size distribution is shown in Figure 1A.

### Cell Viability

THP-1 cells were exposed to Control or PPARγ-expressing microparticles (MP) for 4 hours before activation with LPS or PAM3CSK4. Sixteen hours later cells were stained with the viability dye, AlamarBlue (Invitrogen), and fluorometric values were measured after 10 hours on the Varioskan Flash (Thermo Scientific).

### Flow Cytometry

Cells were fixed in 1% paraformaldehyde and when necessary, permeabilized with Perm Buffer III (558050, BD Biosciences, San Jose, CA), and blocked with FcR Blocking Reagent (130-059-901, Miltenyi Biotec, San Diego, CA). Antibodies used in flow cytometry analysis were: biotin mouse anti-human CD49e (555616, BD Biosciences) with streptavidin-APC (SA1005, Caltag, Buckingham, MK18 1TF), alexa fluor 647 mouse anti-fibronectin (563098, BD Biosciences), alexa fluor 700 mouse anti-human CD19 (557921, BD Biosciences), APC-Cy7 mouse anti-human CD11b (560914 BD Biosciences), PE mouse anti-human CD3 (9515-09, Southern Biotechnology, Birmingham, AL), APC mouse anti-human CD36 (561822, BD Biosciences). For lipid uptake analysis, HCS LipidTOX Red Neutral Lipid Stain (Invitrogen), Low Density Acetylated Lipoprotein from Human Plasma conjugated with Alexa Fluor 594 (Invitrogen). Cells were analyzed on the LSR II (BD Biosciences) or Accuri C6 (BD Biosciences) flow cytometers. Data were analyzed with FLOWJO software (Treestar, Ashland, OR, USA).

### ELISAs

IL-8 sandwich ELISAs were performed with mouse anti-human monoclonal capture and mouse anti-human monoclonal biotinylated detection antibodies (M-801, M-802-B; Endogen, VWR, Radnor, PA). MCP-1 sandwich ELISAs were performed with mouse anti-human monoclonal capture and goat anti-human polyclonal biotinylated detection antibodies (MAB679, BAF279; R&D Systems, Minneapolis, MN). Streptavidin-conjugated alkaline phosphatase (170-3554; BioRad, Hercules, CA) followed by pNPP/DEA solution (37620, Pierce, VWR) were used for colorimetric detection of the cytokines.

### Western Blot

Culture derived microparticles were lysed and 30 µg of protein was run on an 8% polyacrylamide gel, transferred onto PVDF membrane, and verified for equal loading with total protein stain: 0.1% Ponceau S (Sigma) in 0.1% glacial acetic acid (EMD Chemicals; Merck KGaA, Darmstadt, Germany), and 5% glacial acetic acid to de-stain. After blocking in 5% Bovine Serum Albumin (Sigma), membranes were probed with (1∶1000) rabbit anti-human peroxisome proliferator-activated receptor-γ (PPARγ) (clone D69; Cell Signaling Technology, Boston, MA). After 4 washes, the goat anti-rabbit IgG-HRP conjugated secondary antibody (111-035-144, Jackson, West Grove, PA) was added and washed, followed by detection with Immobilon Western Chemiluminescent HRP Substrate (Millipore, Billerica, MA) captured with film (LPS Inc., Rochester, NY).

### Quantitative Real Time PCR

Cells were washed in PBS and lysed with QIAzol Lysis Reagent (Qiagen Sciences, Maryland 20874) and total RNA was extracted with the miRNeasy Mini Kit (217004, Qiagen Sciences) according to manufacturer's instructions. Next, 100 ng of RNA was used to synthesize cDNA using the iScript cDNA Synthesis Kit (170-8891, BioRad), qPCR reactions were set up with appropriate primers (sequences listed in Table 1). All primers were purchased from Integrated DNA Technologies, Inc. (Coralville, Iowa) except GAPDH primers (Invitrogen). Reactions also contained SSoAdvanced SYBR Green Supermix (172-5264, BioRad), and samples were analyzed on the CFX Connect Real-Time System (BioRad) with Bio-Rad CFX Manager 3.1 software. Starting quantity values were normalized to GAPDH for graphing and analyses. For the mRNA profiling studies, a custom PrimePCR plate was designed with 88 inflammatory and adhesion-related targets (BioRad), and the assays were performed according to the manufacturer's instructions.

**Table 1:** Primer sequences.

Gene target	Sequence
Fibronectin Fwd	CCACTTCCCCTTCCTATACAAC
Fibronectin Rev	ACTGATCTCCAATGCGGTAC
ITGA5 Fwd	ATACTCTGTGGCTGTTGGTG
ITGA5 Rev	CTGTTCCCCTGAGAAGTTGTAG
ITGAV Fwd	TGTGCCCCATTGTACCATTG
ITGAV Rev	ACGATTTCTGCCACTTGATCC
MMP1 Fwd	GCACAAATCCCTTCTACCCG
MMP1 Rev	TGAACAGCCCAGTACTTATTCC
CX3CR1 Fwd	AGGCATGGAAGTGTTCTGAG
CX3CR1 Rev	CTAGTCAGCATCAGGTTCAGG
LXRa Fwd	TCCTTTTCTGACCGGCTTC
LXRa Rev	GATGAATTCCACTTGCAGCC
TF Fwd	CCAGAGTTCACACCTTACCTG
TF Rev	CATTCACTTTTGTTCCCACCTG
GAPDH fwd	AGGTGAAGGTCGGAGTCAAC
GAPDH rev	TGGGTGGAATCATATTGGAAC

### Statistical Analyses

All experiments were repeated at least three times using triplicates, with the exception of the gene study RNA transcript comparison plots. Displayed graphs are representative of one experimental replicate. Statistics were performed using the Graphpad Prism 4.0 software. Specific statistical analyses and post tests are indicated in their respective figure legends. All p values<0.05 are indicated with a (*), and other relationships can be assumed as not statistically significant. Error bars represent standard error of the mean.

## Results

### Characterization of engineered microparticles

Culture-derived microparticles were isolated from two transduced Meg-01 cell cultures: 1) GFP-positive Meg-01 (Control MP), and 2) GFP-positive, PPARγ over-expressing Meg-01 (PPARγ MP). In order to test whether culture-derived particles were within the standard size distribution limit of 0.1–1 µm that is typically assigned to microparticles [11], [12], we analyzed vesicle hydrodynamic diameter using dynamic light scattering. Indeed, these culture-derived microparticles had hydrodynamic diameters between 0.1–1 µm (Figure 1A), verifying their microparticle identity and purity. PPARγ protein of microparticle lysates was analyzed by western blot, showing that Control MP had no detectable PPARγ, while PPARγ MP contained high levels of PPARγ (Figure 1B). Increasing the expression of any transcription factor could directly or indirectly alter the transcriptome of the megakaryocytes and the released microparticles. Therefore, we were interested to characterize the RNA profile within the microparticles that resulted from this overexpression. A qPCR profiling array was used to compare mRNA composition of Control vs. PPARγ MP populations (Figure 1C). Multiple targets were differentially expressed between the two populations, and Figure 1D highlights some of the most differentially expressed mRNAs. PPARγ and FABP4 mRNA levels were undetectable in Control MPs, but highly expressed in PPARγ MPs. Interestingly, PPARγ MPs also had higher levels of other transcripts, such as matrix metallopeptidase 11 (MMP11) and the vitronectin-binding integrin (ITGAV). Contrastingly, PPARγ MPs demonstrated decreased transcript levels of matrix metallopeptidase 10 (MMP10), plasminogen activator inhibitor type 1 (SERPINE1), and monocyte chemotactic protein-1 (MCP-1), including undetectable levels of CD40L compared to Control MPs. The differences in mRNA expression supported an important concept that levels of other mediators besides PPARγ are different between the two microparticle groups.

### Activation of THP-1 cells enhances microparticle uptake

We first established that these GFP-expressing, engineered microparticles can bind and be internalized by human THP-1 monocytic cells (Figure 1E). Before testing for functional influences of the engineered microparticles on recipient cells, we determined if the activation of target cells influences the uptake of microparticles. THP-1 cells were stimulated with either the synthetic bacterial mimetic PAM3CSK4, or the diacylglycerol mimetic phorbol myristate acetate (PMA) during a 16 hour exposure of sub-saturating levels of microparticles. Activation with these stimuli caused a dose-dependent increase in GFP-expressing microparticle internalization compared to unactivated THP-1 cells (increase from 33 to 60%; Figure 1F), potentially enhancing the functional impact of microparticles on these cells.

### Identification of PBMC subsets that internalize microparticles

**Figure 2:**
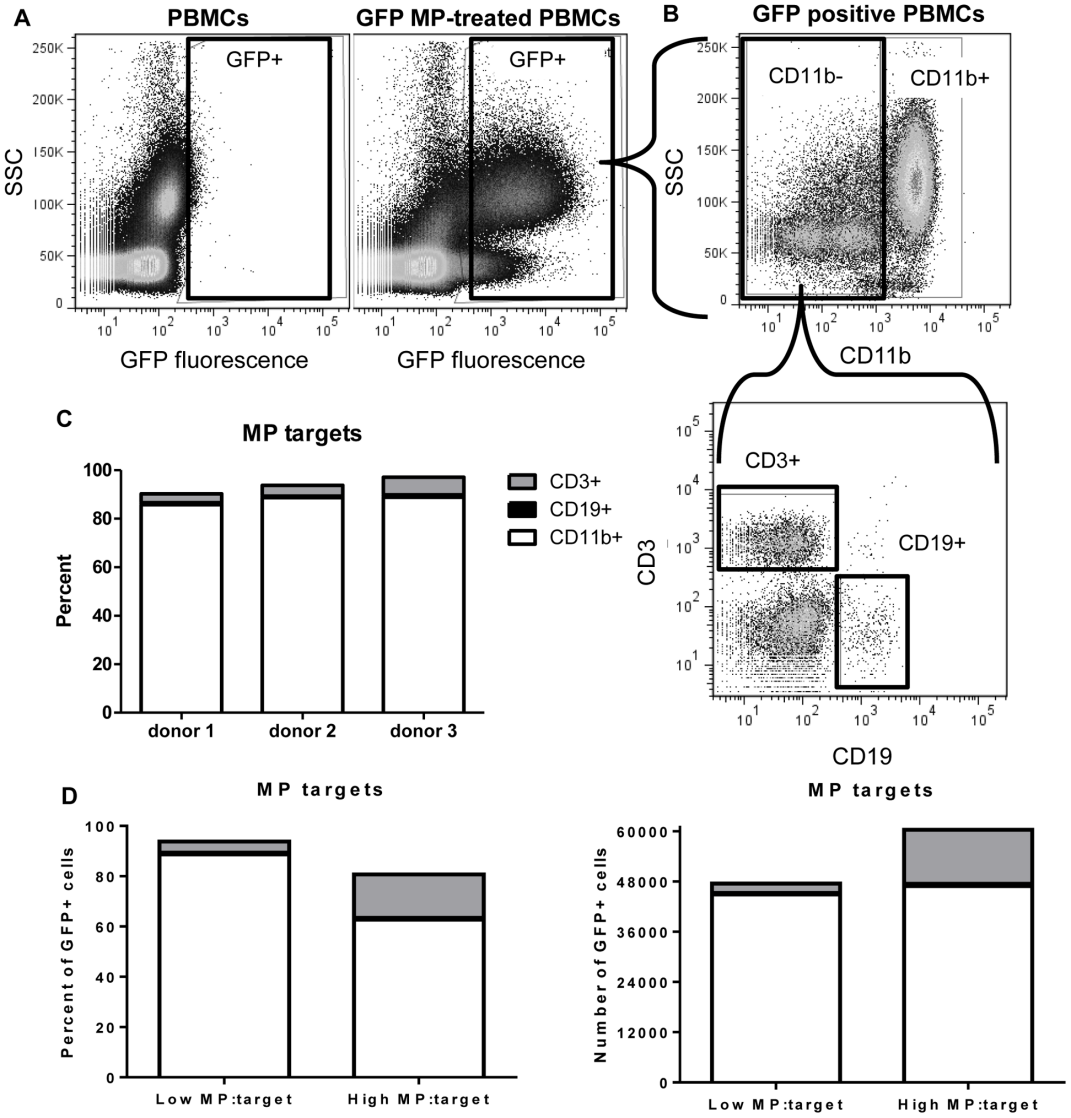
Culture-derived microparticles (MP) are primarily internalized by monocytes among the peripheral blood mononuclear cells (PBMC) populations. PBMCs were isolated and cultured for 24 hours with green fluorescent protein (GFP)+ microparticles, and then washed, stained, and analyzed by flow cytometry. **A**, Cells that internalize or bind to GFP+ microparticles (right) exhibit green fluorescence compared to cells not treated with microparticles (left). **B**, Cells were gated on GFP and then subgated on CD11b, followed by CD3 and CD19 to identify specific cell types. **C**, PBMCs from three representative donors demonstrate that the majority of the GFP positive cells are positive for CD11b (white boxes). **D**, Ten times more microparticles were added to PBMCs (High MP∶target) to identify the any other subpopulation to internalize microparticles. One representative donor PBMC demonstrates that CD3+ cells (grey boxes) were the second cell type to internalize microparticles according to percentage (left) and numbers (right) of GFP-positive PBMCs.

In addition to utilizing a widely used human monocytic cell line (THP-1), it was important to test if engineered microparticles could be internalized and influence the function of primary human blood cells. We first set out to discover which blood cell type, if any, would bind and/or internalize culture-derived microparticles. Peripheral blood mononuclear cells (PBMCs) were isolated from healthy human donors and incubated with GFP-expressing microparticles for 24 hours. Cells were then washed, stained, and analyzed by flow cytometry. The three major populations of PBMCs are T lymphocytes, B lymphocytes, and monocytes, which were labeled with anti-CD3, CD19, and CD11b antibodies, respectively. GFP+ PBMCs were sub-gated based on their staining pattern into one of the three aforementioned populations (Figure 2A&B). PBMCs from three different donors internalized GFP+ microparticles in a similar pattern, with the CD11b+ monocytes acting as the predominant population that internalized microparticles (Figure 2C), despite the fact that CD11b+ cells only comprised 10–20% of total PBMCs. This preference of microparticle internalization by monocytes over other cell types has not been previously known. Additionally, it was consistent that >90% of the total CD11b+ cells internalized microparticles (pre-gated on CD11b+, post gated on GFP+; data not shown). We further asked what other, if any, cell subsets also internalize microparticles. To this end, we exposed the same number of PBMCs to two doses of GFP-expressing microparticles: “Low MP: target” vs. “High MP: target” (difference of 10-fold). In both groups, the same number of CD11b+ cells internalized microparticles, indicating that this population was likely the most efficient at internalizing microparticles. According to GFP+ numbers and percentages of PBMCs, CD3+ cells were the next best population to bind and internalize microparticles (Figure 2D). However, in an effort to minimize the complexity of a multicellular culture and, as monocytes showed the strongest ability to internalize microparticles, we chose to move forward and characterize the functional influence of engineered culture-derived microparticles on purified human monocytes.

### Monocyte mRNA changes after microparticle exposure

**Figure 3:**
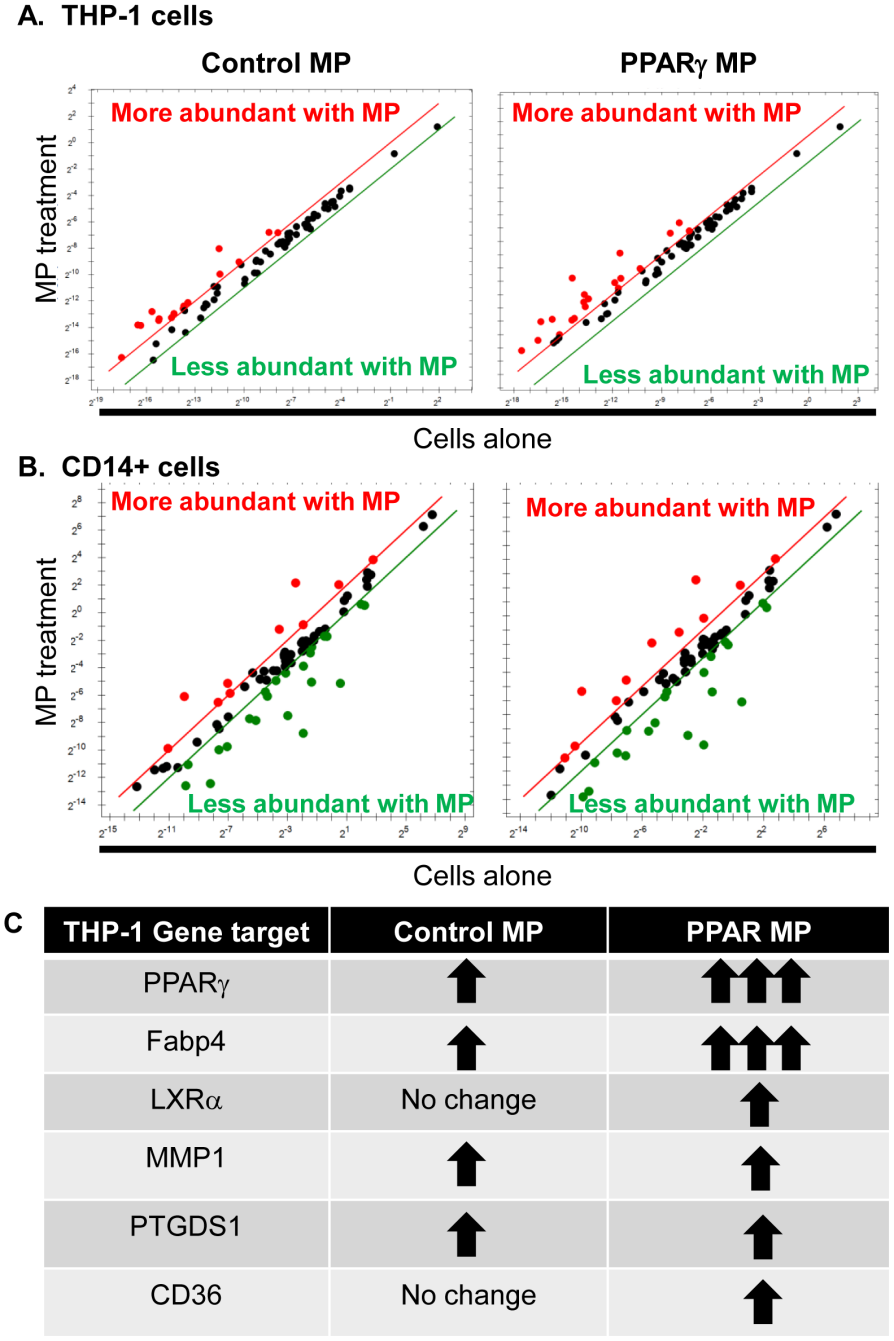
Monocyte mRNA expression is influenced by microparticle composition. THP-1 cells (**A**) or CD14 sorted blood monocytes (**B**) were cultured with Control MP (left) or PPARγ-containing MP (PPARγ MP; right) for 24 hours. Cells were washed, lysed, and mRNA was isolated for qPCR analysis. Diagonal thresholds were drawn to depict transcript levels that changed more than two-fold. Red and green dots outside of the diagonal lines represent transcripts that are upregulated or downregulated, respecitively, with the addition of microparticle treatment compared to THP-1 cells alone. **C**, Select upregulated transcripts of interest were validated by qPCR in at least three independent experiments using THP-1 cells. Arrows represent relative change of transcript expression with microparticle exposure compared to unexposed cells.

We next wanted to discover the important recipient cell transcripts that were affected by the composition of PPARγ MP. We cultured Control or PPARγ MP with unactivated THP-1 cells or purified CD14+ blood monocytes for 24 hours. Cells were then washed and lysed for RNA isolation used in an mRNA profiling array. Both types of monocytes had changes in several target transcript levels after 24 hour exposure to either microparticle population (Figure 3A&B). We further verified changes in select mRNA target levels by conventional qPCR from THP-1 cells incubated with either Control or PPARγ MP (Figure 3C). PPARγ and FABP4 were highly induced with PPARγ MP treatment compared to THP-1 cells alone. Additionally, CD36 and Liver X Receptor-α (LXRα) (other downstream targets of PPARγ were also increased with PPARγ MP treatment, further validating effective PPARγ delivery and activity inside of the target cells. Interestingly, several transcript levels involved in monocyte maturation or thrombotic potential, such as integrin-α_M_ (CD11b), CD40, and P-selectin glycoprotein ligand-1 remained unchanged in the microparticle-exposed monocytes (data not shown).

### Activated monocytes have altered proinflammatory cytokine production dependent on microparticle composition

**Figure 4:**
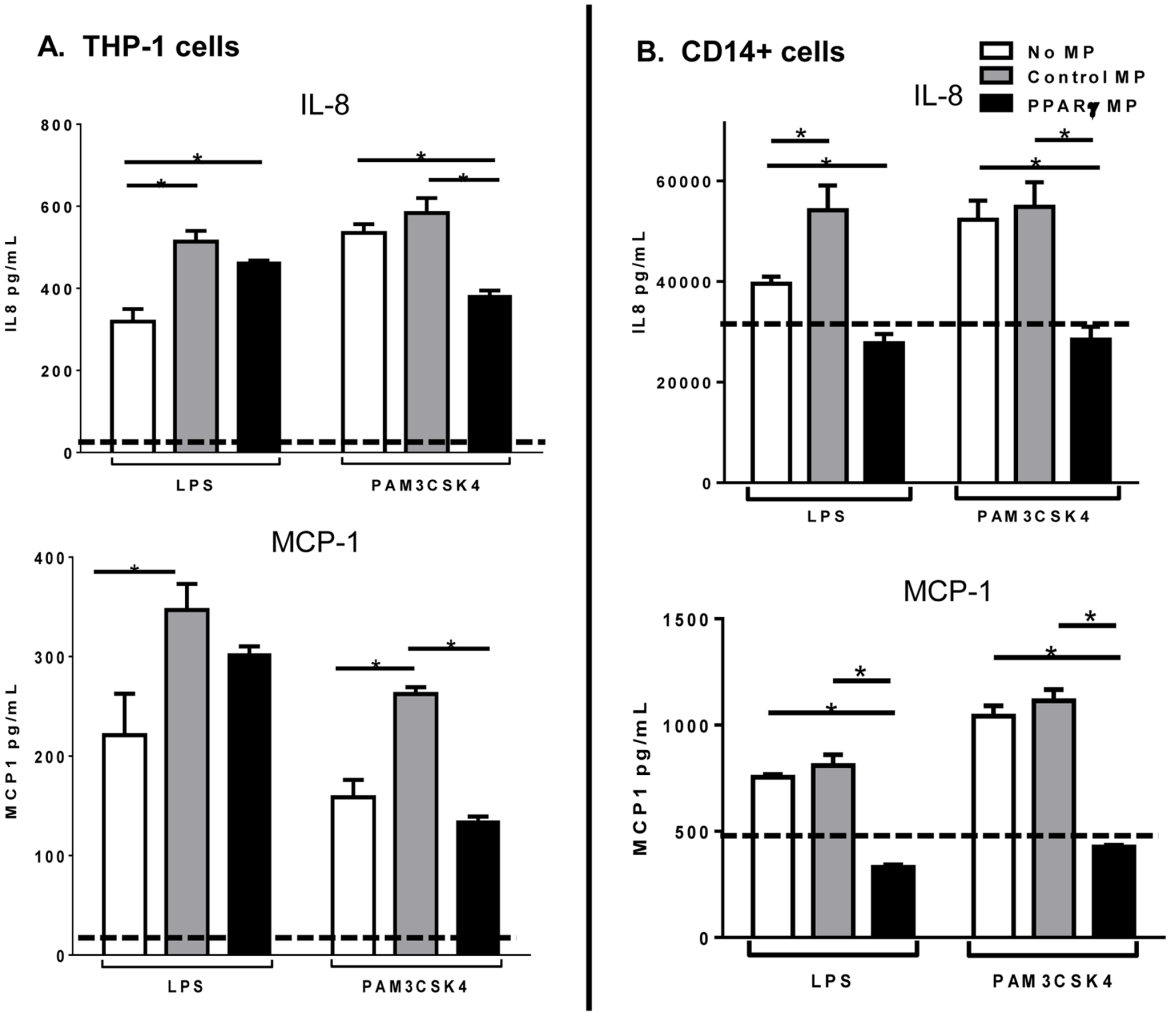
Microparticle composition influences inflammatory cytokine production from activated monocytes. THP-1 cells (**A**) or CD14 sorted blood monocytes (**B**) were treated with control microparticles (grey bars) or PPARγ-overexpressing microparticles (black bars) or no microparticles (white bars) for 4 hours before activation with LPS or PAM3CSK4 for 24 hours. Supernatants were collected and pro-inflammatory cytokines IL-8 (top), MCP-1 (bottom) were measured by ELISA. Dotted line represents baseline cytokine production from unactivated cells with no microparticle exposure. Mean values with standard errors represent one of at least 3 experiments. Two-way ANOVA with Tukey's multiple comparison post test was performed to determine statistical significance. * indicates (p<0.05).

PPARγ expression by macrophages is reported to have anti-inflammatory properties [13]. We therefore tested if treatment with PPARγ MP would influence the inflammatory response of THP-1 cells and primary human monocytes. After a four hour exposure to microparticles, low doses of bacterial mimetics, LPS or PAM3CSK4 were added as potent proinflammatory activators for 24 hours. Cell culture supernatants were then collected and the proinflammatory mediators interleukin (IL)-8 and monocyte chemotactic factor-1 (MCP-1) were measured via ELISA. Microparticles themselves can induce proinflammatory responses from exposed cells independent of other activators [14]. This trend was observed when Control MP increased proinflammatory cytokine production over activated cells alone. Excitingly, PPARγ MP treatment did not induce the same increase in inflammatory cytokine production as Control MP, and in some cases even decreased cytokine production below that of activated cells alone (Figure 4). Overall, the internal composition of the microparticles influenced how monocytes responded to proinflammatory stimulation.

In addition to inflammatory-related changes of monocytes, we tested whether microparticles could influence the production of a key thrombotic player, tissue factor. Unactivated THP-1 cells exposed to Control MP had increased levels of tissue factor expression over PPARγ MP-treated cells or cells alone (Figure S2). Activation of the cells without microparticles increased tissue factor expression of cells alone. However, both microparticle-exposed groups of activated cells did not exhibit any increases or differences in tissue factor expression.

To address whether cytokine or mRNA expression level differences in microparticle treated cells were due to changes in viability, we treated and activated THP-1 cells in the same manner as Figure 4, and measured viability with the AlamarBlue dye (Figure S3). Microparticle composition did not affect cell viability in activated or unactivated cells.

### Monocyte adherence changed dependent on microparticle composition

**Figure 5:**
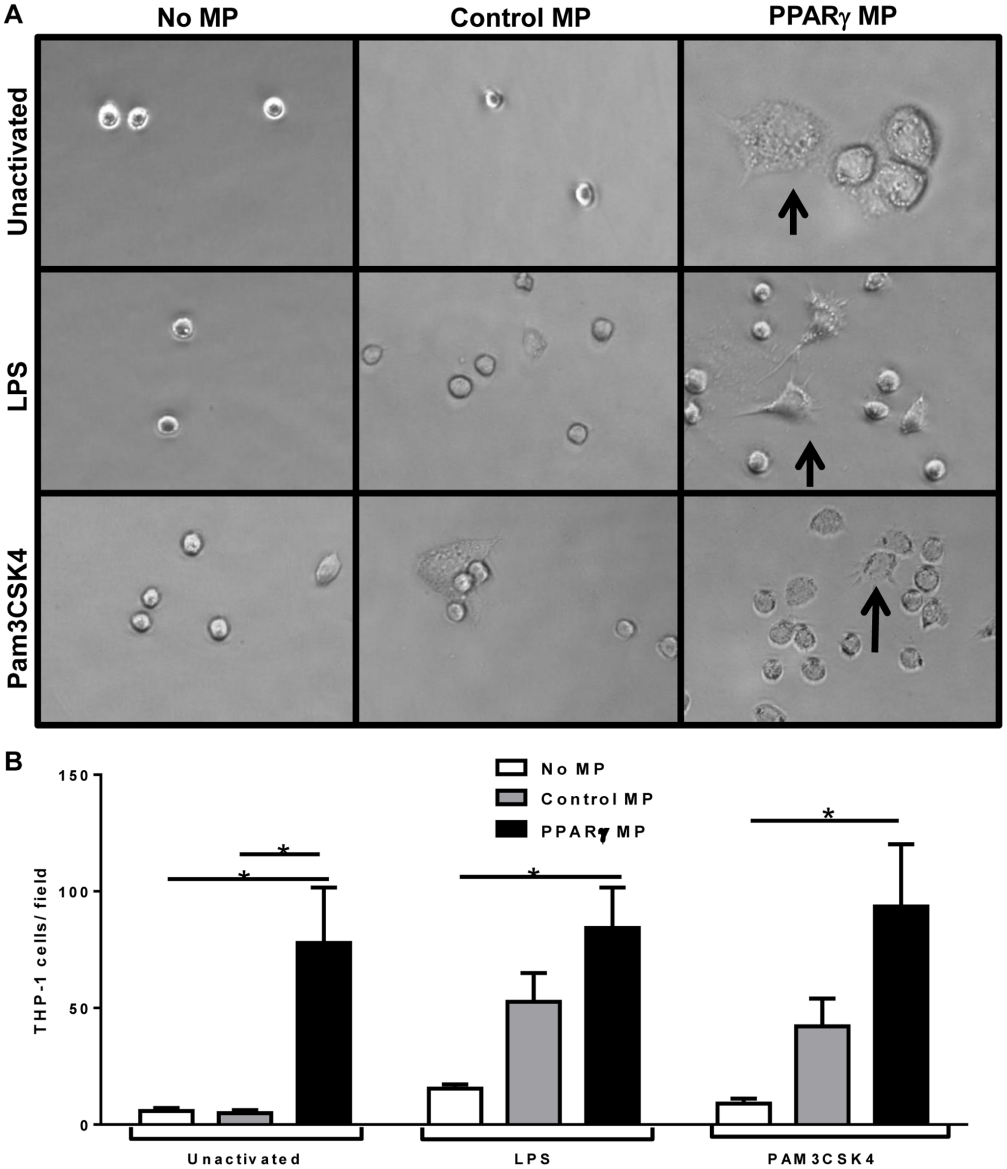
PPARγ-containing microparticles (PPARγ MP) increase adherence and spreading of THP-1 cells, but not lipid uptake. THP-1 cells were treated with microparticles for 4 hours and left unactivated or activated with LPS or PAM3CSK4 for 24 hours. Cells were removed from culture wells, which were then washed with phosphate buffered saline. **A**, Cells that were still attached to the wells were visualized with the 40× objective lens of an inverted microscope, and representative images from at least three independent experiments are shown. Of note, some of the PPARγ MP treated cells that were attached had a morphology indicative of spreading (arrows). **B**, Attached cells from five fields of view in each condition were quantified, and PPARγ MP treated cells always contained the most adherent cells. Two-way ANOVA with Tukey's multiple comparison post test was performed to determine statistical significance. Mean values with standard errors averaged from 5 experiments. * indicates (p<0.05).

During MP treatment of activated THP-1 monocytes, we were surprised to observe striking morphologic differences between treatment groups. Unlike purified CD14+ monocytes, which uniformly adhere to tissue culture plastic upon plating, THP-1 monocytes are typically uniformly suspension cells. Interestingly, PPARγ MP-treated THP-1 cells adhered much more to the tissue culture plate compared to control groups. To quantify this, we first exposed THP-1 cells to microparticles for four hours before addition of either media alone, LPS or PAM3CSK4 for 24 hours. After the 24 hour treatment, non-adherent cells were removed by aspirating the culture medium and washing the plates three times in phosphate buffered saline. The remaining adherent cells were visualized with an inverted microscope (Figure 5A). In addition to an increased number of attached cells, many of the adherent cells from the PPARγ MP treatment groups had a “spread” type of morphology. The numbers of adherent THP-1 cells that were exposed to Control or PPARγ MP and left unactivated or activated with LPS or PAM3CSK4 were quantified. In all conditions, PPARγ MP treatment induced the most adhesion (Figure 5B).

PPARγ, monocyte adherence and lipid uptake have roles in atherosclerosis [15], [16]. Therefore, we wanted to address whether the adherent cells from any of the aforementioned conditions were accumulating more lipid than others. We visualized lipids within fixed adherent cells via Oil-Red-O staining, and imaged the slides with differential interference contrast microscopy (Figure S4A). All conditions contained THP-1 cells with lipid vacuoles. To further test if PPARγ overexpression may cause increases of lipid uptake, THP-1 cells were directly transduced with PPARγ-expressing lentivirus, which could be detected with fluorescence from the GFP reporter. 50% of non-transduced cells and 50% PPARγ-transduced cells were plated in the same well, and AlexaFluor 594-conjugated acetylated low density lipoprotein was added to the culture for 24 hours before the cells were removed, washed and analyzed on flow cytometry (Figure S4B). All cells demonstrated ability to take up low density lipoprotein, regardless of PPARγ expression. Lastly, primary CD14+ cells were treated with either microparticle group to determine influences of lipid uptake. After 24 hours of microparticle exposure, cells were harvested for analysis via flow cytometry (Figure S4C). Analysis included the Class B scavenger protein involved in lipid uptake (CD36) and a fluorescent lipid stain. Mean fluorescence intensities demonstrate that PPARγ MP exposure did not increase CD36 expression or lipid uptake. Overall, all of the tested monocytes readily internalized lipids, and microparticle treatments did not impact lipid uptake.

### Monocytes treated with PPARγ MP upregulate adhesive molecules

**Figure 6:**
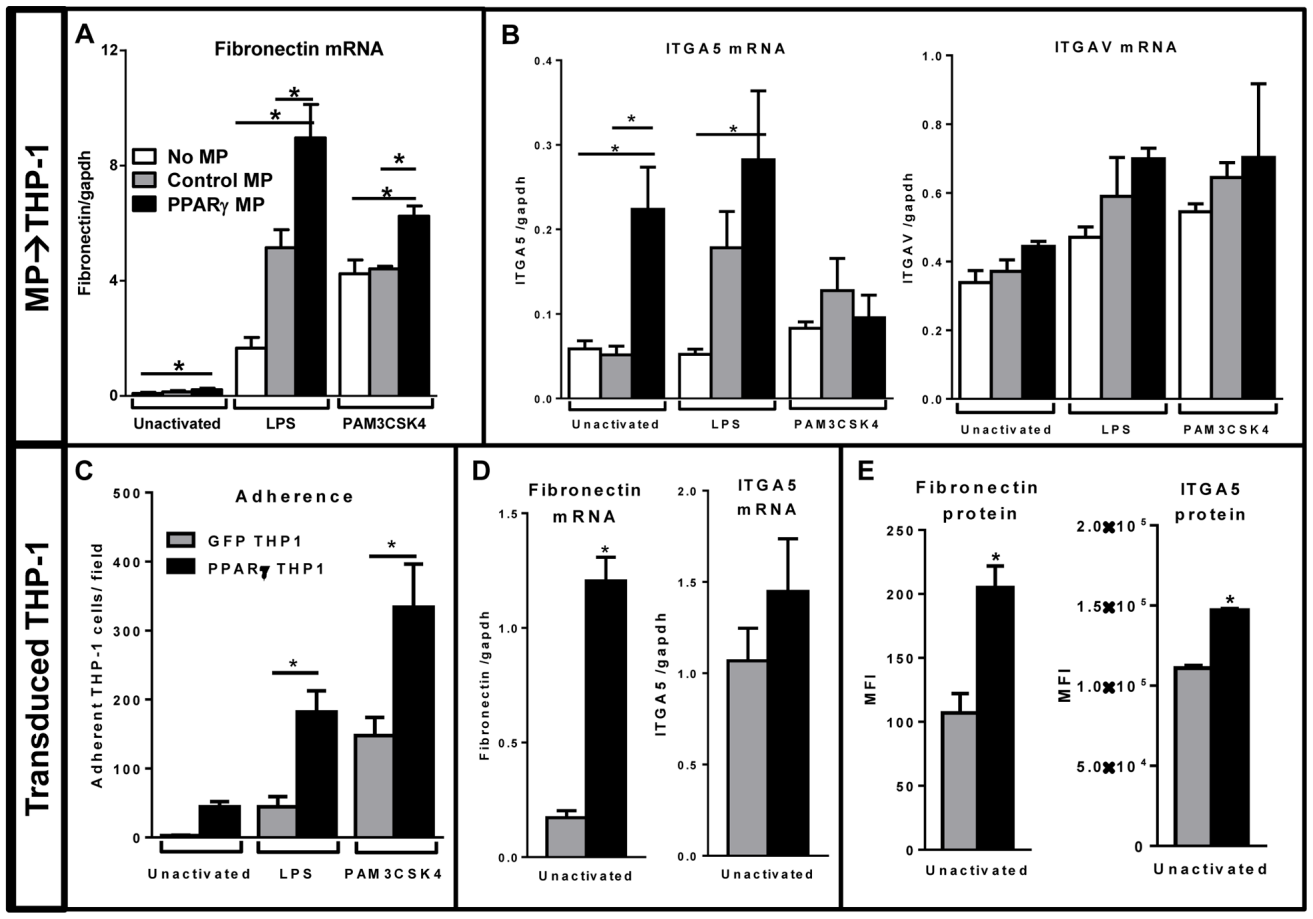
PPARγ-containing microparticles (PPARγ MP) or direct transduction of PPARγ increase fibronectin production and ITGA5 expression of THP-1 cells. **A–C**, THP-1 cells were treated with Control MP (grey bars), PPAR MP (black bars), or no microparticles (white bars) for 4 hours before activation with LPS or PAM3CSK4 for 24 hours. Cells were washed and lysed. RNA was isolated and fibronectin levels were measured via qPCR (**A**), indicating PPARγ MP increased fibronectin expression from activated cells. **B**, Integrins ITGA5 and ITGAV were also measured by qPCR, showing THP-1 cells treated with PPARγ MP had the highest expression of the fibronectin receptor, ITGA5, but no significant changes were seen with the vitronectin receptor, ITGAV. **C–E**, THP-1 cells were directly transduced with a control (GFP THP1; grey bars) or a PPARγ-overexpressing lentivirus (PPARγ THP1; black bars) and expanded for one week. Cells were then plated and left unactivated, or stimulated with LPS or PAM3CSK4 for 24 hours at which point suspension cells were removed and the wells were washed three times in phosphate buffered saline. **C**, Adherent cells were visualized and counted with an inverted microscope, and cells which overexpressed PPARγ demonstrated increased adherence compared to the control groups. Unactivated transduced THP-1 cells were lysed for mRNA (**D**) and protein (**E**) analysis. THP-1 cells that overexpress PPARγ had significant upregulation of fibronectin mRNA and protein as well as ITGA5 protein. Mean values with standard errors represent one of at least 3 experiments. Two-way ANOVA with Tukey's multiple comparison post test (**A–C**), or Student's T Test (**D–E**) were performed to determine statistical significance. * indicates (p<0.05).

To further investigate the mechanism behind PPARγ MP-induced adhesion of THP-1 cells, we investigated the expression of molecules known to be involved in monocyte adhesion. Monocytes roll and adhere to atherosclerotic lesions via fibronectin through VLA4 integrin expression [17]. Additionally, monocyte binding to fibronectin via another integrin, VLA5 (ITGA5), results in pro-angiogenic activity [18]. Therefore, we investigated fibronectin mRNA levels in unactivated, LPS-, or PAM3CSK4-activated THP-1 cells after treatment with microparticles. In step with the observed adhesion patterns (Figure 5B), we saw that activation of THP-1 cells increased expression of fibronection mRNA (Figure 6A). Moreover, fibronectin mRNA expression was further upregulated in both activation groups receiving PPARγ MP.

It was noteworthy that while fibronectin was being synthesized at low levels by unactivated THP-1 cells (Figure 6A), another source of fibronectin could be from the serum in the culture medium. Adhesion to fibronectin requires expression of fibronectin-binding integrins, therefore, we investigated changes in receptors involved in adhesion. Takizawa et al. showed monocytes bound and were activated by fibronectin via ITGA5 [19]. We thus tested if expression of the specific fibronectin-binding integrin, ITGA5, was altered with PPARγ MP exposure. Excitingly, PPARγ MP treatment caused a significant increase of ITGA5 mRNA in the unactivated and LPS-activated THP-1 groups. This change was specific for the fibronectin-binding integrin, as the vitronectin-binding integrin, ITGAV, had no significant changes among any treatment condition (Figure 6B).

### Direct overexpression of PPARγ in THP-1 cells increases adherence

PPARγ MPs taken up by THP-1 cells change the cells adherence properties (Figure 5). This phenotype could be due to several possible mechanisms, such as signaling of surface receptors or through the transfer of the PPARγ transcription factor, itself. To test if the microparticle delivery of PPARγ was mediating this phenotype, and to further prove the importance of PPARγ in the THP-1 cell adhesion, we tested whether fibronectin and its integrin levels could be upregulated if PPARγ was directly overexpressed in THP-1 cells. Using lentiviral gene delivery, we overexpressed PPARγ (PPARγ THP-1) or a GFP-control (GFP THP-1) directly in the THP-1 cells. After one week of expansion, equal numbers of cells were plated into a tissue culture plate and left unactivated or activated with LPS or PAM3CSK4 for 24 hours, after which, non-adherent cells were aspirated, the wells were washed three times in phosphate buffered saline, and attached cells were imaged and quantified with an inverted microscope. In all conditions, PPARγ THP-1 cells were more adherent than control THP-1 cells (Figure 6C). We also collected total mRNA from unactivated cultures to quantify levels of fibronectin and the fibronectin binding integrin, ITGA5 (Figure 6D). Compared to the control transduction, THP-1 cells that overexpressed PPARγ had a significant increase in fibronectin levels. To further support the upregulation of these two molecules, we next investigated protein levels in transduced THP-1 cells. Cells were fixed and permeablized for immunostaining, followed by analysis via flow cytometry. GFP+ events were pre-gated, and the mean fluorescence intensity (MFI) of fibronectin or ITGA5 protein, minus unstained background values, were analyzed (Figure 6F). Both fibronectin and ITGA5 protein expression were significantly increased in THP-1 cells that overexpressed PPARγ.

## Discussion

The level of blood microparticles is increased and in some cases their composition is altered in diseases involving vascular inflammation [6]. Microparticles can signal surface receptors, or even transfer surface proteins to recipient cells to impact their function [11]. However, little is known about how the internal composition of microparticles affects transcellular communication. We therefore used a novel approach to investigate the functional influence of two engineered types of microparticles, specifically in an inflammatory setting. We have shown that microparticles that contain the anti-inflammatory transcription factor PPARγ decrease monocyte proinflammatory cytokine production of IL-8 and MCP-1 and increase cell adherence. These results provide important fundamental validation that the composition of microparticles influences the inflammatory responses of recipient cells. Conceivably, an increase of proinflammatory mediators, such as certain transcription factors within microparticles would perpetuate vascular inflammation upon transfer to target cells. Therefore, evaluating the total content of blood microparticles as biomarkers for inflammatory disease status is an attractive diagnostic platform.

We demonstrated that THP-1 monocytes have an increased capacity for microparticle internalization after cell stimulation (Figure 1F). Activation of THP-1 cells possibly caused an increase of microparticle internalization due to increased phagocytosis, but this mechanism of uptake was not verified. Alternative explanations include surface expression changes on THP-1 cells after stimulation that may bind and internalize microparticles more efficiently.

The preference of microparticle interaction among a mixed population of blood cells has not been previously studied. We showed CD11b+ monocytes were the most dominant of the various PBMC subsets to internalize microparticles (Figure 2). Interestingly, T cells also stood out among the subsets that demonstrated ability to interact with microparticles. It is notable that B cells, albeit low in frequency among PBMCs, also displayed the ability to interact with microparticles. T and B cells have not been previously shown to internalize microparticles in the presence of monocytes. Therefore, these data provide new verification that circulating microparticles are capable of interacting and influencing many different cell types *in vivo*. The mechanism of interaction and internalization of microparticles was not investigated here, but would be important to understand how these microparticles communicate with each cell type.

We report herein that the total compositional differences between Control and PPARγ MPs affected recipient cell function. Despite the differences we detected in microparticle composition (Figure 1D), our data strongly support PPARγ expression and delivery as the driving force between the different responses in the recipient cells. In further support of this concept, we directly overexpressed PPARγ within the monocytes and observed the same phenotype induced by the PPARγ microparticles (Figure 6). To better classify PPARγ-specific effects in future studies, inhibition of PPARγ activation with a specific antagonist could be tested in the future.

With a complete blockade of PPARγ function in recipient cells, we may identify other components of the microparticle populations that contribute to altered function in recipient cells. The mRNA comparison of the two microparticle groups indicated that Control MPs contain more CD40L and MCP-1 mRNA than PPARγ MPs (Figure 1D), exposing possible signaling mechanisms other than PPARγ delivery to explain how these microparticles increased proinflammatory mediator production over the activated PPARγ MP-treated cells, or activated cells alone.

This work was performed without exogenous addition of PPARγ ligands in order to limit other variables within our study and highlight the sole influences of different microparticle composition on recipient cell function. PPARγ-driven effects shown in this report could be independent of ligand activation or initiated by endogenous PPARγ ligands such as 15-deoxy-prostaglandin-J_2_ introduced in culture serum or produced by the recipient cells. Further expansion of the investigations in this system with various doses and durations of pharmacologic PPARγagonists and/or inhibitors would be of interest, especially those with clinical relevance, such as the thiazolidinedione class of anti-diabetic PPARγ ligands.

Importantly, PPARγ MP exposure to either primary human monocytes or THP-1 monocytes caused decreased levels of proinflammatory mediator release compared to Control MP treatments (Figure 4), thus validating that total microparticle composition affects monocyte inflammatory responses. It is noteworthy that our supernatant ELISA or cell lysate RNA analyses of monocyte function involved the cumulative response of all cells. Single cells or multiple populations within the treated monocytes may be affected differently than the sum of the recipient cells. Future studies should include flow cytometry analysis to tease out potential subpopulations that may be differentially affected by the microparticles.

PPARγ activation has been shown to induce cell differentiation [16], [20]. Ricote et al. demonstrated that PPARγ was upregulated during thioglycolate-elicited mouse monocyte differentiation into macrophages [13]. Therefore, in agreement with work in other systems, our results support the idea that increased expression of PPARγ in monocytes, whether through microparticle delivery or direct overexpression, increased monocyte differentiation leading to enhanced cell adhesion. This could have implications *in vivo*, where microparticle delivery of PPARγ could cause monocytes to adhere to atherosclerotic plaques or initiate monocyte diapedesis across the endothelium. However, *in vivo* testing of monocytes would be necessary to show the relevance of the phenotypic changes exhibited by PPARγ MP exposed monocytes. If microparticle-delivered PPARγ could induce cell differentiation while dampening inflammation in an *in vivo* system, it would support microparticle delivery as an attractive future therapeutic.

Microparticle exposures of activated blood monocytes resulted in similar effects on cytokine release compared to THP-1 cells. However, the two types of monocytic cells have some differences. Primary CD14+ sorted cells adhere to tissue culture plastic within hours [20], whereas THP-1 cells remain in suspension until a stimulus is added [21]. Unactivated primary monocytes had basal cytokine production levels near half of those treated with LPS and PAM3CSK4, yet unactivated THP-1 cell produced almost negligible amounts of IL-8 and MCP-1 cytokines. These observations led us to infer that the isolation process for primary CD14+ human monocytes by positive selection created enough of a stimulus to lightly activate these cells and possibly cause them to commence maturation and differentiation into macrophages. Therefore, changes in adherence of primary monocytic cells were not observed in these culture conditions, as the cells were already uniformly adherent prior to microparticle exposure. THP-1 cells, however, exhibited striking differences in adherence based on microparticle treatment and activation (Figure 5). The ability of microparticles to induce an adherent cell phenotype is supported by the work of Nomura et al., which demonstrated that platelet-derived microparticles enhanced adhesion-related surface receptor expression on THP-1 and endothelial cells [14]. Importantly, our work showed that this increase of adhesion is dependent on microparticle composition. Specifically, microparticle delivery of PPARγ promoted cell adhesion, similar to THP-1 cells that were directly transduced with a PPARγ-expressing lentivirus(Figure 6).

We show that PPARγ MP treatment increased fibronectin mRNA expression and its cognate binding integrin, ITGA5, in recipient THP-1 cells. These key proteins are likely playing a major role in THP-1 cell adhesion, although we cannot rule out involvement of other unidentified mediators. Interestingly, monocyte ITGA5 binding to fibronectin was shown to induce monokine production of IL-1, TNFα, and IL-6 by Takizawa et al. [19]. This phenotype could also be occurring in our model of monocyte activation, however, the complexities and kinetics of this proinflammatory signaling mixed with the anti-inflammatory influences of PPARγ MP (Figure 4) would need to be further elucidated. Adding further intricacy, Takizawa et al. declared that their “results suggest that conformational change or fragmentation of fibronectin is essential for the activation of monocytes through VLA-5” [ITGA5] [19]. It is likely that the amount and conformation of fibronectin present in the environment of monocytes will determine the strength of the signaling they receive, leading to adhesion, activation or both. With these factors in mind, matrix degradation and remodeling proteins would be interesting future targets to pursue in further exploration of fibronectin signaling in monocytes.

An alternative explanation for the induction of ITGA5 expression is that PPARγ MP are inducing a pro-angiogenic “myeloid-endothelial” phenotype as described by Li et al. [22]. These investigators found TNFα-stimulated monocytes upregulated ITGA5, causing a significant increase in adhesion to fibronectin-coated coverslips. In our studies, we did not pre-coat any of the tissue culture wells, rather fibronectin was produced by the cells themselves and also could have come from serum in cell culture media. We have not performed analysis on endothelial markers to identify if they are entering a transition towards a myeloid-endothelial phenotype, but this is plausible and worthy of further investigation.

In conclusion, we propose that delivery of PPARγ from microparticles initiates monocyte adherence and possibly differentiation towards a macrophage or “myeloid-endothelial” cell phenotype, but one which is less responsive to inflammatory stimuli than those receiving control microparticles. This work is the first to demonstrate how microparticle-contained transcription factors can be transferred to cells to influence their function. The delivery of the anti-inflammatory transcription factor, PPARγ, provided a particularly powerful influence within recipient cells, as PPARγ can initiate transcription of several downstream products to influence multiple pathways. Our novel system to engineer microparticle composition permits a new understanding of microparticles and transcellular communication. Furthermore, this technology may provide a platform for the engineering of microparticles for future investigations of therapeutics and delivery of anti-inflammatory mediators to an inflamed vasculature.

## Conclusions

The evaluation of human blood samples for microparticle number, cell source, surface and internal composition has been proposed to provide invaluable biomarkers to assist in diagnosis of inflammatory conditions. Here, we demonstrate that internal composition of engineered microparticles affects target cell inflammatory responses. Activated monocyte exposure to engineered microparticles with high levels of the anti-inflammatory transcription factor, PPARγ, resulted in decreased proinflammatory mediator production compared to cells treated with control microparticles. Additionally, microparticles expressing PPARγ induced THP-1 monocyte adherence, possibly reflecting differentiation influences. Taken together, these results provide important, new, fundamental validation that the total composition of microparticles influences recipient cell function. Considering their validated influences, microparticles could conceivably be used as clinical diagnostic biomarkers of circulating inflammation, or engineered to deliver therapeutic mediators in future medical practices.

## Supporting Information

Figure S1 **Nanodrop spectrophotometry is more sensitive than the BCA protein assay and provides a linear correlation for relative quantification of serial diluted microparticles.** A suspension of intact microparticles was prepared and serially diluted 2-fold in RPMI1640. Measurements given as “protein concentration,” referred to here as arbitrary units, were determined via nanodrop (blue diamonds) or Bicinchoninic Acid protein assay (BCA; Red boxes) methods. Values minus the RPMI1640 blank control were graphed and a trendlines were drawn with equations and correlation coefficients shown. The BCA assay was unable to detect the 4 lowest concentrations of microparticles (red Xs), and for the purposes of this comparison, were graphed at values of zero. Unlike the BCA assay, the nanodrop measurements were able to measure the full dilution range of microparticles, and importantly, did so in a linear relationship allowing for accurate comparison and normalization of microparticle populations in linear proportion equations.
(TIF)

Figure S2 **Tissue Factor mRNA is induced by activation of THP-1 cells but not microparticle exposure.** THP-1 cells were exposed to Control or PPARg-expressing microparticles (MP) for 4 hours before activation with LPS or PAM3CSK4. 24 hours later cells were harvested and mRNA was analyzed with qPCR. Unactivated cells exposed to Control, but not PPARg-expressing microparticles had a slight increase of tissue factor expression. Activation of cells without microparticle exposure increased tissue factor expression, however, both microparticle-exposed cells did not exhibit any increase of tissue factor. Data are shown of technical replicates from one out of two representative experiments. Data were analyzed with Two-way ANOVA and Tukey's multiple comparison post test. * indicates (p<0.05).
(TIF)

Figure S3 **Microparticle exposure enhanced cellular metabolism, but microparticle composition did not impact viability.** THP-1 cells were exposed to Control or PPARg-expressing microparticles (MP) for 4 hours before activation with LPS or PAM3CSK4. Sixteen hours later cells were given the viability reagent, AlamarBlue (Invitrogen), and fluorometric values were measured after 10 hours on the Varioskan Flash (Thermo Scientific). Two-way ANOVA with Tukey's multiple comparison post test was performed to determine statistical significance. * indicates (p<0.05) Biological replicates from one representative out of two experiments are shown.
(TIF)

Figure S4 **Neither microparticle composition nor direct PPARg overexpression affected lipid uptake of monocytes.** **A**, THP-1 cells were treated in wells on a 8-well chamber slide (Millipore, Billerica, MA), cultured with no microparticles (no MP), GFP microparticles (GFP MP) or PPARg-containing microparticles (PPARg MP) for 24 hours. Afterwards, the wells were washed twice with PBS, fixed in 3% paraformaldehyde (Electron Microscopy Sciences, Hatfield, PA), washed again in PBS and then covered with the lipid stain, Oil-Red-O [60% Oil Red O in isopropanol diluted in water; 0.2 um filtered] (Cayman Chemical Company, Ann Arbor, Michigan). The solution was incubated on a rotating rocker for 10 minutes, washed twice with distilled water, mounted with coverslip and then images were taken using differential interference contrast microscopy. Representative images are shown in all conditions, indicating all cells had similar uptake and storage of lipids. **B**, To further test if PPARg overexpression may cause increases of lipid uptake, THP-1 cells were directly transduced with PPARg-expressing lentivirus, which could be detected with fluorescence from the GFP reporter. 50% of non-transduced cells and 50% PPARg-transduced cells were plated in the same well, and 25 mg/mL of AlexaFluor 594-conjugated acetylated low density lipoprotein was added (LDL; red) to the culture for 24 hours before the cells were removed, washed and analyzed on flow cytometry. Compared to cells that did not receive LDL (blue), all cells demonstrated similar LDL uptake (y-axis), regardless of PPARg expression. **C**, Primary CD14+ monocytes were isolated from human blood and treated with no MP, GFP MP or PPARg MP for 96 hours. All cells were washed and stained with 1∶500 Lipidtox Red and with an antibody for the Class B scavenger protein involved in lipid uptake (CD36) for analysis via flow cytometry. Frequency and mean fluorescent intensity (MFI) of CD36 staining, and MFI of lipid fluorescence from all CD14+ cells (left) or gated cells that have taken up GFP fluorescent microparticles (right) are listed. Frequency of lipid+ cells was 100% in all conditions, therefore lipid MFI is listed to indicate quantity of lipid in the cells. All data shown are from individual experiments that have been repeated at 3 times.
(TIF)
