# Supplementary figures and images for: Microparticles Engineered to Highly Express Peroxisome Proliferator-Activated Receptor-γ Decreased Inflammatory Mediator Production and Increased Adhesion of Recipient Monocytes
Source: PLoS One. 2014 Nov 26;9(11):e113189. doi: 10.1371/journal.pone.0113189 (PMC4245109; doi:10.1371/journal.pone.0113189)

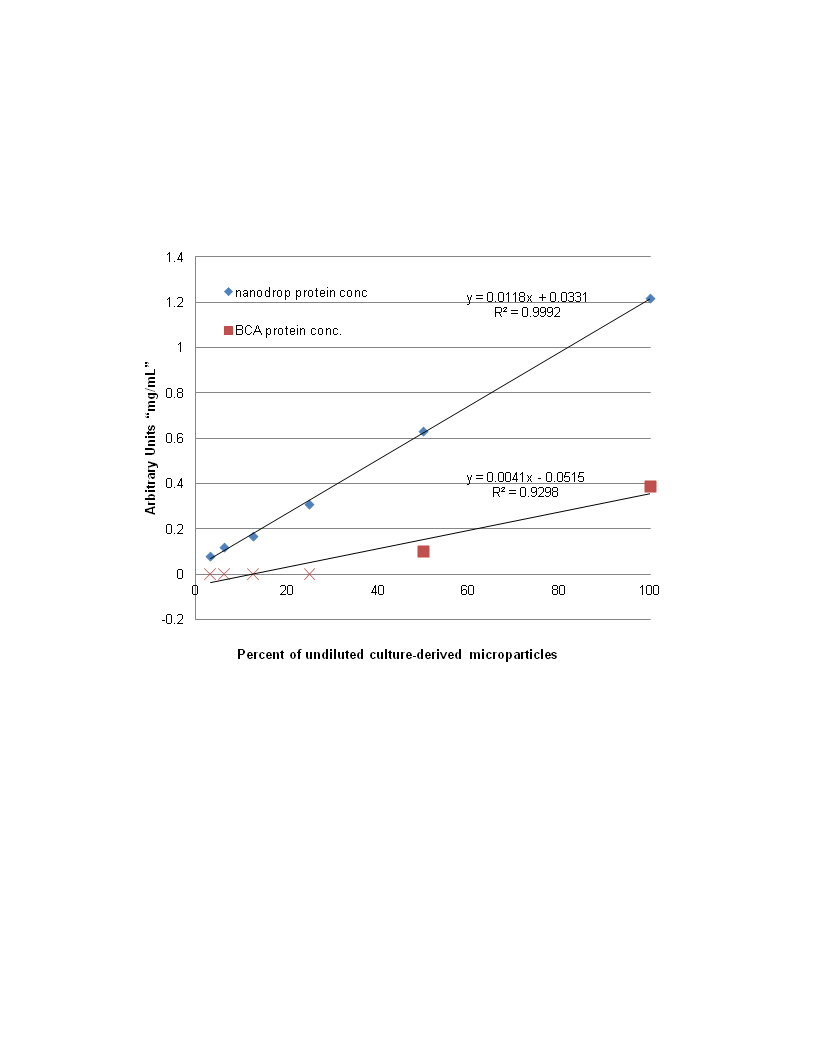

Supplement: Figure S1 — Nanodrop spectrophotometry is more sensitive than the BCA protein assay and provides a linear correlation for relative quantification of serial diluted microparticles. A suspension of intact microparticles was prepared and serially diluted 2-fold in RPMI1640. Measurements given as “protein concentration,” referred to here as arbitrary units, were determined via nanodrop (blue diamonds) or Bicinchoninic Acid protein assay (BCA; Red boxes) methods. Values minus the RPMI1640 blank control were graphed and a trendlines were drawn with equations and correlation coefficients shown. The BCA assay was unable to detect the 4 lowest concentrations of microparticles (red Xs), and for the purposes of this comparison, were graphed at values of zero. Unlike the BCA assay, the nanodrop measurements were able to measure the full dilution range of microparticles, and importantly, did so in a linear relationship allowing for accurate comparison and normalization of microparticle populations in linear proportion equations. (TIF) [file pone.0113189.s001.tif]

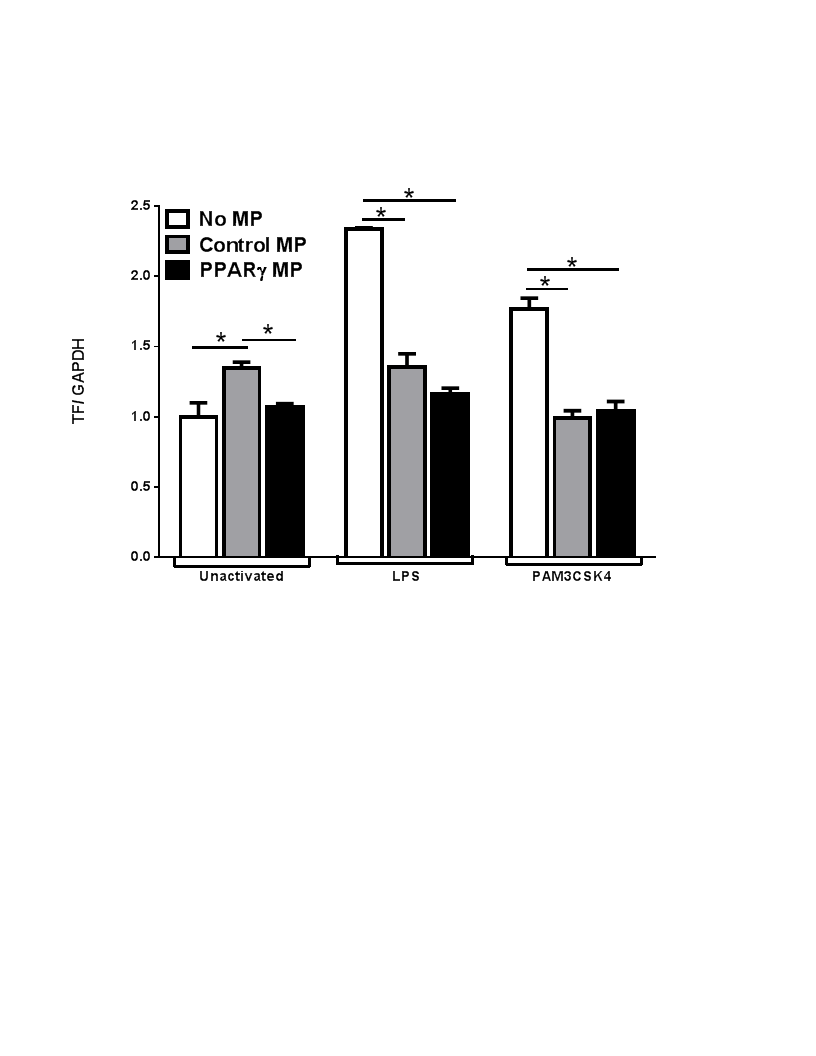

Supplement: Figure S2 — Tissue Factor mRNA is induced by activation of THP-1 cells but not microparticle exposure. THP-1 cells were exposed to Control or PPARg-expressing microparticles (MP) for 4 hours before activation with LPS or PAM3CSK4. 24 hours later cells were harvested and mRNA was analyzed with qPCR. Unactivated cells exposed to Control, but not PPARg-expressing microparticles had a slight increase of tissue factor expression. Activation of cells without microparticle exposure increased tissue factor expression, however, both microparticle-exposed cells did not exhibit any increase of tissue factor. Data are shown of technical replicates from one out of two representative experiments. Data were analyzed with Two-way ANOVA and Tukey's multiple comparison post test. * indicates (p<0.05). (TIF) [file pone.0113189.s002.tif]

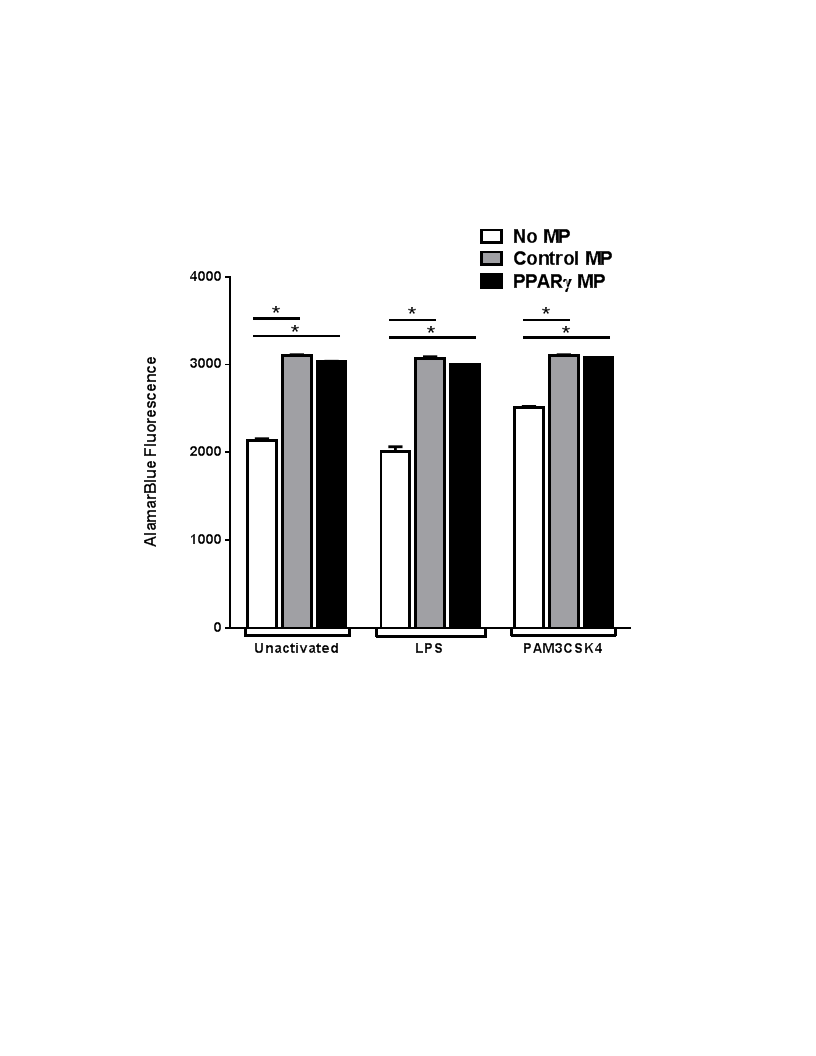

Supplement: Figure S3 — Microparticle exposure enhanced cellular metabolism, but microparticle composition did not impact viability. THP-1 cells were exposed to Control or PPARg-expressing microparticles (MP) for 4 hours before activation with LPS or PAM3CSK4. Sixteen hours later cells were given the viability reagent, AlamarBlue (Invitrogen), and fluorometric values were measured after 10 hours on the Varioskan Flash (Thermo Scientific). Two-way ANOVA with Tukey's multiple comparison post test was performed to determine statistical significance. * indicates (p<0.05) Biological replicates from one representative out of two experiments are shown. (TIF) [file pone.0113189.s003.tif]

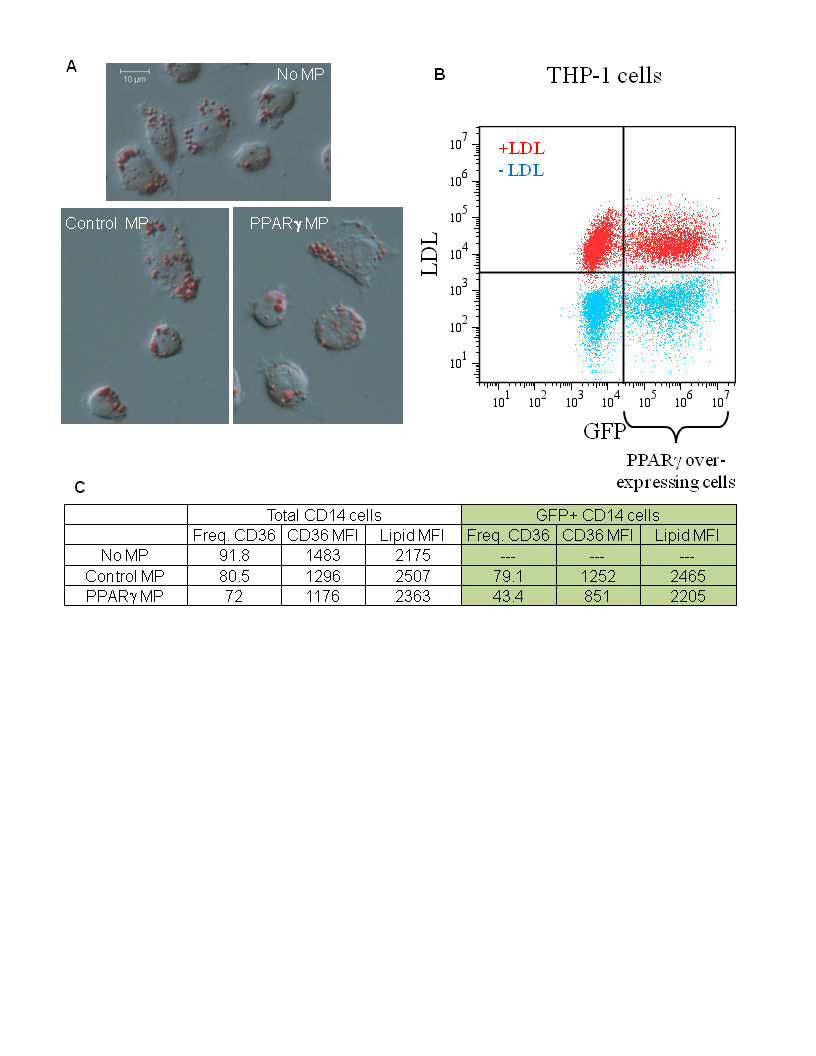

Supplement: Figure S4 — Neither microparticle composition nor direct PPARg overexpression affected lipid uptake of monocytes. A, THP-1 cells were treated in wells on a 8-well chamber slide (Millipore, Billerica, MA), cultured with no microparticles (no MP), GFP microparticles (GFP MP) or PPARg-containing microparticles (PPARg MP) for 24 hours. Afterwards, the wells were washed twice with PBS, fixed in 3% paraformaldehyde (Electron Microscopy Sciences, Hatfield, PA), washed again in PBS and then covered with the lipid stain, Oil-Red-O [60% Oil Red O in isopropanol diluted in water; 0.2 um filtered] (Cayman Chemical Company, Ann Arbor, Michigan). The solution was incubated on a rotating rocker for 10 minutes, washed twice with distilled water, mounted with coverslip and then images were taken using differential interference contrast microscopy. Representative images are shown in all conditions, indicating all cells had similar uptake and storage of lipids. B, To further test if PPARg overexpression may cause increases of lipid uptake, THP-1 cells were directly transduced with PPARg-expressing lentivirus, which could be detected with fluorescence from the GFP reporter. 50% of non-transduced cells and 50% PPARg-transduced cells were plated in the same well, and 25 mg/mL of AlexaFluor 594-conjugated acetylated low density lipoprotein was added (LDL; red) to the culture for 24 hours before the cells were removed, washed and analyzed on flow cytometry. Compared to cells that did not receive LDL (blue), all cells demonstrated similar LDL uptake (y-axis), regardless of PPARg expression. C, Primary CD14+ monocytes were isolated from human blood and treated with no MP, GFP MP or PPARg MP for 96 hours. All cells were washed and stained with 1∶500 Lipidtox Red and with an antibody for the Class B scavenger protein involved in lipid uptake (CD36) for analysis via flow cytometry. Frequency and mean fluorescent intensity (MFI) of CD36 staining, and MFI of lipid fluorescence from all CD14+ cells (left) or [file pone.0113189.s004.tif]
